# Editorial: Invasive fungal diseases: pathogen detection and diagnosis development

**DOI:** 10.3389/fcimb.2024.1473409

**Published:** 2024-09-17

**Authors:** Ying Zhao, Qinning Wang, Patrick C. Y. Woo

**Affiliations:** ^1^ Department of Clinical Laboratory, Peking Union Medical College Hospital, Chinese Academy of Medical Sciences, Beijing, China; ^2^ Microbial Genomics Reference Laboratory, Centre for Infectious Diseases and Microbiology Laboratory Services, Institute of Clinical Pathology and Medical Research, NSW Health Pathology, Sydney, NSW, Australia; ^3^ Doctoral Program in Translational Medicine and Department of Life Sciences, National Chung Hsing University, Taichung, Taiwan; ^4^ The iEGG and Animal Biotechnology Research Center, National Chung Hsing University, Taichung, Taiwan

**Keywords:** invasive fungal infection, diagnosis, *Candida*, mold, progress

Advancement of medical care, such as the exponential expansion of the use of biologics, has resulted in an unprecedented number of immunocompromised patients who are prone to various kinds of opportunistic fungal infections that are associated with significant mortality and morbidity (Chan et al., 2015; Li et al., 2020). Successful management of these fungal infections require a high index of suspicion, rapid and accurate laboratory diagnosis and prompt commencement of antifungal treatment. Traditionally, laboratory diagnosis of fungal infections was achieved through direct detection in clinical samples by microscopic examination of potassium hydroxide smear for fungal hyphae and Indian ink stain smear for *Cryptococcus neoformans*, isolation of the fungal organism and identification using biochemical tests and microscopy. However, these methods are associated with a number of difficulties, such as slow or difficult-to-grow fungi and the requirement of expertise who are capable to recognize the different microscopic features of molds, as the traditional way of identifying this group of filamentous fungi involves extracting morphological data and compares them with the classical images in textbooks. Other methods, such as antigen and antibody detection, are also used. For example, the (1-3)-β-D glucan test is used in some centers (Zhao et al., 2022), but this method has a major drawback of being non-specific.

Polymerase chain reaction (PCR) amplification and sequencing of the ITS1-5.8S-ITS2 rRNA gene cluster of the fungal genome, often referred to as ITS sequencing, is extremely useful for rapid and objective identification of many groups of fungi (Zhao et al., 2018). However, for certain genera where several closely related fungal species may share almost identical ITS sequences, amplification and sequencing of additional housekeeping gene loci, such as the translation elongation factor 1-alpha (TEF-1α) gene, β-tubulin gene and calmodulin gene, may be necessary (Woo et al., 2008; Tsang et al., 2020); with the choice of additional gene target(s) depending on the genus of the fungus. In this Research Topic, a number of studies have used ITS sequencing as well as sequencing of other housekeeping gene loci for identification of fungal pathogens. In one report, Erami et al. have employed ITS and TEF-1α sequencing to investigate three cases of *Fusarium* rhinosinusitis during the COVID-19 pandemic, in which they found *F. proliferatum*, *F. oxysporum* and *Aspergillus flavus*, and *F. solani/falciforme* to be the culprits of the *Fusarium* infections. In another report, Aboutalebian et al. used ITS sequencing to identify the first case of *Candida palmioleophila* candidemia in an infant with biliary atresia in Iran. In a third study, Rouhi et al. used a two-step multiplex PCR coupled with DNA sequencing for analysis *Candida* species, including *Candida auris*, isolated in respiratory samples from patients with COVID-19 infections.

In the last decade, next-generation sequencing (NGS) technologies have been used more and more widely for laboratory diagnosis of infectious diseases. For fungal organisms, the most commonly detected one is *Pneumocystis jirovecii*, followed by *Aspergillus* species, *Candida* species, *Cryptococcus* species, etc (Tsang et al., 2021; Xing et al., 2024). Different NGS platforms have their own advantages and disadvantages. Short-read sequencers, such as the Illumina platform, are best known for their low sequencing error rates and costs; whereas the Oxford Nanopore Technologies’ MinION device has gained popularity because of its low equipment cost, short turn-around-time, portable size, and scalability. In this Research Topic, a number of studies have demonstrated the usefulness of NGS for laboratory diagnosis of fungal diseases, analysis of antifungal resistance genes, and evaluating the effect of fungal infection on the gut microbiota. In a case of pleural infection that Jia et al. encountered with thymoma and myasthenia gravis on long-term corticosteroid and tacrolimus treatment, NGS analysis of the pleural biopsy sample confirmed the identity of *C. neoformans* and hence a rare case of pleural cryptococcosis. In another report, Hong et al. have employed NGS whole-genome sequencing and identification of resistance genes in a patient with urinary catheter-related *C. auris* urinary tract infection. In a third study, Yan et al. examined the influence of pathogenic *C. albicans* on the gut microbiota using an immunodeficient mouse model.

Apart from PCR amplification, DNA sequencing and NGS, this Research Topic also includes a number of other studies that use various novel methods to improve diagnosis of fungal infections. In one study, Liu et al. used (1-3)-β-D glucan mutant antibody for chemiluminescence detection of (1-3)-β-D glucan as an alternative to Limulus amebocyte lysate, which overcome the problem of the scarcity of Limulus resources for (1-3)-β-D glucan. In another study, Ye et al. designed a rapid duplex flap probe-based isothermal assay to identify *C. neoformans* and *C. gattii*, the two species complexes associated with cryptococcosis. In a third study, Li et al. used advanced mathematical modelling and statistical methods to predict invasive fungal super-infections during healthcare-associated bacterial infections in the intensive care unit, which will facilitate the development of specific risk-based targeted and timely prevention and control measures. In the next decade, we anticipate that the number of fungal infections diagnosed by these *state-of-the-art* technologies will continue to increase in an exponential manner. For example, the most crucial limiting factor for the widely use of NGS in laboratory diagnosis is cost. When the cost of NGS is further reduced and expertise more widely available, routine use of NGS for laboratory diagnosis of fungal infections would not be a dream anymore.
